# A Novel Tool Condition Monitoring Technique of Determining Insert Flank Wear Width of Indexable Face Milling Tools Using On-Machine Laser Tool Setters

**DOI:** 10.3390/mi16101169

**Published:** 2025-10-15

**Authors:** Tao Fang, Zezhong Chen, Haibo Feng, Peng Chen, Zhiyong Chang

**Affiliations:** 1 Department of Mechanical Engineering, Northwestern Polytechnical University, Xi’an 710072, China; fangtnwpu@mail.nwpu.edu.cn (T.F.); elijahchern@mail.nwpu.edu.cn (P.C.); 2 Institute for Aero-Engine Smart Assembly of Shaanxi Province, Xi’an 710072, China; 3 Zhejiang Advanced CNC Machine Tool Technology Innovation Center Co., Ltd., Taizhou 317503, China; 4 Gina Cody School of Engineering and Computer Science, Concordia University, Montreal, QC H3G 1M8, Canada; louisfenghaibo@gmail.com

**Keywords:** on-machine tool measurement, tool radius measurement, tool flank wear width, tool replacement, smart machining

## Abstract

Indexable face milling tools are often used to machine workpieces with large axial and radial depth of cuts, and thus, the inserts quickly wear out in machining. A kernel technique of smart machining is tool wear compensation, which is to regularly and automatically measure the insert radius/length with a laser tool setter on the machine table during machining, and compensate them in the subsequent machining. Another technique is tool condition monitoring, which is to calculate the insert flank wear width for tool condition and compare with its threshold. When it is less than but close to its threshold of invalid inserts, the cutting tool is automatically changed right before it becomes invalid. On-machine laser tool setters have been equipped in CNC machine tools for several years; however, they cannot conduct cutting tool condition monitoring. The main reason is that the insert flank wear width cannot be measured on the on-machine laser tool setter, and the status quo is that the cutting tool is replaced either too early or too late. To address this problem, a novel tool condition monitoring technique of determining the insert flank wear width of indexable face milling tool using on-machine laser tool setters is proposed. According to the insert geometry, the worn cutting edge and a new workpiece milling mechanism proposed in this work, the insert flank wear width can be calculated. In machining, the insert radius wear is measured on the on-machine laser tool setter, and the insert flank wear width is calculated to evaluate whether it is invalid soon. The results indicate that the optimal height for radius measurement is located near the intersection of the corner and side edges point *MR*_3_, and close to the cutting depth point *MR*_5_. A wear land width threshold of 0.10 mm is established to define tool failure. The proposed calculation method achieves high accuracy, maintaining calculation errors within 14.00%. The inserts can be used in good condition with the maximum lifespan. This method has been verified in machining applications and can be directly applied in industry.

## 1. Introduction

A kernel technique of smart (or intelligent) machining is on-machine tool measurement for tool wear compensation and tool condition monitoring. Specifically, radii and lengths of the cutting tools should be regularly, automatically, and precisely measured with the laser tool setter on the machine table; no machinist is needed. After measuring the cutting tool, its radius and length wear is compensated in the subsequent machining so that the workpiece accuracy remains high as before. Tool wear compensation is realized. At the same time, the tool wear amount is calculated and compared to its threshold so that it can be automatically changed right before it becomes invalid. Therefore, the tool can effectively cut qualified workpieces with its best longevity, and the tool cost is reduced. Tool condition monitoring is realized. However, in the conventional machining, machinists do not regularly measure the tool radius and length with manual tool setters outside the CNC machines during machining; the tool wear compensation cannot be conducted. When mechanists physically notice the tool wears out, they pause machining and manually measure the tool to determine whether to replace the tool or not. This tool condition monitoring is ineffective. It is in high demand in industry to conduct the tool wear compensation and the tool condition monitoring in machining.

Tool condition monitoring is critical to workpiece quality and the maximum extent of using tools [1]. During the machining process, the cutting tool continuously removes material from the workpiece and gradually undergoes wear. The wear pattern of the tool is influenced by numerous factors, primarily including cutting parameters (cutting speed, feed rate, and depth of cut), tool material and geometry (carbide and coatings), workpiece material and geometry, machine tool conditions, and the cooling methods (dry machining, minimum quantity lubrication, and minimum quantity cooling lubrication) employed [2]. Tool wear patterns are typically categorized into several types, including flank wear, crater wear, plastic deformation, notch wear, thermal cracking, chipping, and built-up edge (BUE) [3]. It has been found that the main wear pattern for coated cemented carbide tools when cutting steel materials is flank wear [4]. During machining, the flank face of the tool remains in continuous contact with the machined surface of the workpiece. This contact generates significant frictional heat on the workpiece surface, which leads to gradual wear on the flank face [5].

The influence of tool wear on cutting forces is significant and cannot be ignored. Gao et al. [6] developed a cutting force prediction model that comprehensively considers tool flank wear and tool runout. Milling experiments showed that as the flank wear increases, the engagement between the tool and the workpiece becomes greater, resulting in a sharp rise in cutting forces. Ding et al. [7] constructed a real-time cutting force monitoring model that integrates tool deformation, tool runout, and tool wear. They proposed a tool wear prediction method based on temporal convolutional networks, bi-directional long short-term memory networks, and multi-objective optimization algorithms, achieving a maximum prediction error of 12.02%. Wang et al. [8] considered the impact of tool wear on the actual milling cutter radius and developed a stainless steel milling force prediction model that accounts for tool wear. The experimental results show that during the rapid wear stage, the milling force generated by a worn tool is more than twice that of a new tool. Song et al. [9], aiming to reveal the mechanism by which vibration affects cutting force and tool wear in ultrasonic-assisted machining, established a two-dimensional ultrasonic-assisted scratching force model that incorporates tool wear, confirming the inhibitory effect of ultrasonic vibration technology on tool wear. A worn cutting tool leads to progressive deterioration of the cutting edge, which can result in “chip-like burr” forming on the workpiece surface, severely affecting surface quality [10]. As is well known, tool wear directly determines both tool life and workpiece machining quality. Worn cutting tools increase the surface roughness of the workpiece and reduce machining accuracy. As tool wear intensifies, both the tool diameter and length continue to decrease. The purpose of tool condition monitoring is to measure the reduction in tool dimensions and thereby calculate the extent of tool wear.

Face milling is one of the primary types of milling processing and is widely used in the machining of large components [11]. Face milling involves high material removal rates and significant energy consumption, which generates substantial cutting heat and leads to severe tool wear [12]. The wear distribution on the tool is non-uniform [13], primarily concentrated near the cutting edge due to the combined effects of extrusion deformation and shear deformation imposed by the tool on the workpiece [14]. Pozzato et al. [15] used round ceramic inserts to machine Inconel 718 superalloy and found that the central region of the insert flank face experienced the most severe tool wear. Zhao et al. [16] developed a non-uniform flank wear model considering the real cutting length (RCL) of the tool, and based on this, proposed a tool orientation optimization method to reduce the maximum flank wear and thereby extend tool life. Jin et al. [17] machined unidirectional CFRP composites using indexable milling tools and observed non-uniform wear distribution caused by the different fiber orientations in the UD layers. Therefore, to accurately measure tool wear and improve measurement efficiency, it is essential to identify the region where maximum tool wear occurs.

As a new technology, on-machine measurement (OMM) enables automatic measurement of tool radius and length during machining by using tool setters mounted on the machine table, allowing for tool path compensation in subsequent processes. Liu et al. [18] developed a non-contact automated tool setter for micro-milling machines using a fiber optic sensor composed of a laser emitter and receiver. The sensor calculates the tool position by detecting the change in light intensity as the tool passes through the beam, and compares it with the beam location to determine the tool dimensions. The system achieved a measurement accuracy of 2 µm and repeatability of 0.6 µm for micro-tools of various sizes, with each measurement taking approximately 10 s, and an 80% improvement in efficiency compared to manual tool setting. In ultra-precision machining, laser diffraction-based micro-tool measurement techniques offer significant application value. Khajornrungruang et al. [19] utilized laser diffraction fringe characteristics to precisely measure the dimensions of rotating micro end mills and drills on the machine tool. The accuracy of tool setter measurements depends on factors such as calibration accuracy and measurement parameters. Fang et al. [20] developed a new calibration method by establishing a laser beam axis calibration mechanism, which accurately determined the position of the laser tool setter in the machine coordinate system and significantly improved its calibration accuracy. Vieira Junior et al. [21] conducted a series of experiments to thoroughly investigate the effects of spindle speed and feed rate on the measurement accuracy of tool dimensions using a laser tool setter. The optimal measurement parameters were determined to achieve precise tool size measurement. Although the above-mentioned laser tool setter can directly measure the tool radius and length, it is incapable of measuring flank wear on the tool and therefore cannot achieve the objective of tool condition monitoring.

Accurately measuring tool wear is a challenging task, especially in finishing processes where tool wear typically occurs on the micron scale, making measurement even more difficult [22]. Moreover, tool wear is inherently uncertain and presents in complex and varied patterns, which adds to the difficulty of tool wear measurement. The measurement methods of tool wear can be classified into two main categories: direct and indirect measurement methods. Direct measurement methods involve directly capturing the geometric features and dimensions of the worn region to assess the wear condition of the tool. This approach primarily focuses on evaluating parameters such as the width [23], area [24], and volume [25] of the tool wear regions. Meanwhile, indirect measurement methods assess tool wear by capturing dynamic signals generated during the cutting process that are correlated with the tool wear condition. These signals include vibration, current and power signals, cutting force, temperature, and acoustic emission, among others [26]. Saha et al. [27] investigated progressive tool wear indicators in micro-milling processes, such as the increase in cutting edge radius, the reduction in tool radius, and the increase in flank wear land width. And established a geometric relationship between the change in cutting edge radius and the corresponding reduction in tool radius. By directly measuring these three wear-related indicators on worn-out tools and conducting an in-depth analysis, they concluded that the reduction in tool radius is the most suitable indicator for plotting tool life curves. Li et al. [28] pointed out that, unlike progressive tool wear, tool breakage is sudden and random, posing a more severe threat to workpiece quality, machine tool integrity, and operator safety. The impact of data imbalance on tool breakage monitoring models is explored, and feasible solutions are proposed at both the data and algorithm levels. Liu et al. [29] proposed a vision-based method for precise measurement of tool geometry and established a multi-zone light source illumination model considering multiple incident angles to improve lighting uniformity. Experimental results show that image uniformity increases by 1.5 times, with a relative measurement error of less than 0.35%. Zhu et al. [30] developed a single-image super-resolution method for on-machine measurement of tool wear. By reconstructing high-resolution tool wear images, the tool wear region is measured accurately, and the goal of online evaluation of tool condition has been achieved. Zhang et al. [31] integrated multiple advanced image processing techniques and proposed a visual measurement method for flank wear volume. Using Accelerated-KAZE (AKAZE) feature matching for image registration, the flank wear region is extracted, allowing a precise assessment of the flank wear condition. Wang et al. [32], under minimum quantity lubrication (MQL) cutting conditions, eliminated interface acoustic emission (AE) burst signals by configuring appropriate AE parameters (including threshold, rise time, and duration time), and performed clustering of AE energy to establish the relationship between flank wear and AE burst signals for accurate assessment of tool flank wear. Akhtar et al. [33] utilized infrared sensors to monitor the machining process, including workpiece deflection, chatter, and tool wear, enabling estimation of tool wear at different machining stages. Cheng et al. [34] designed a multi-level parallel convolutional neural network (parallel CNN) framework based on a bi-directional long short-term memory network (BiLSTM) to monitor tool wear conditions under various working scenarios.

Compared with methods relying on a single sensor, most current studies adopt multi-sensor signal fusion for tool wear monitoring [35]. This approach enables information complementation, overcoming the limitations of single-sensor signals that fail to fully characterize tool wear features, thus improving the accuracy of tool wear monitoring. He et al. [36] investigated a deep learning method based on a Stacked Sparse Autoencoder (SSAE) and multi-sensor signal feature fusion. Cutting force, vibration, and acoustic emission signals collected during milling were analyzed in the time domain, frequency domain, and time-frequency domain, respectively. The most sensitive features were selected through correlation analysis, which were input into the SSAE model for deep feature learning to build a predictive model for tool wear. Sun et al. [37] proposed a hybrid-driven, physics-informed Gaussian process regression model to predict tool wear, primarily using cutting force and vibration signals. The model achieved a prediction accuracy of 99.7%. Liu et al. [38] developed an indirect tool wear monitoring system for online measurement of cutting force and cutting temperature. An analytical model was established to relate the flank wear rate of WC-Co cemented carbide tools to cutting force and temperature during the milling process. Zhang et al. [39] introduced a tool wear monitoring method based on multi-channel hybrid information and deep transfer learning. This method integrates six-dimensional sensor data, including cutting force, acoustic emission, vibration signals, and so on. The features extracted from the multi-channel hybrid information showed stronger correlation with tool wear compared to those from single-channel sensor data.

With the rapid advancement of machine learning technologies, many researchers have recently applied machine learning algorithms to tool condition monitoring (TCM). For instance, Cheng et al. [40] proposed an intrinsic information-constrained unsupervised domain adaptation approach to achieve robust feature extraction and transfer learning of tool wear under varying cutting conditions. Tang et al. [41] developed a hybrid learning model to perform time–sequence prediction based on fused sensor features, thereby improving wear prediction accuracy. Similarly, Sun et al. [42] presented a parallel neural network framework for intelligent tool wear monitoring under variable machining conditions, demonstrating strong adaptability in feature learning and state classification. These studies indicate that machine learning-based approaches can effectively mine latent features from complex signals and capture nonlinear degradation trends, providing valuable support for tool wear prediction and remaining useful life estimation. However, most existing studies primarily focus on tool wear condition classification, such as distinguishing between initial wear, severe wear, and tool breakage, rather than achieving precise wear quantification. In addition, it often requires expensive sensors and strict installation conditions, which limit their applicability in real industrial environments [43].

To address the limitations of existing insert flank wear width cannot be measured on the on-machine laser tool setter, this study proposes a novel tool condition monitoring technique capable of determining the flank wear width of indexable face milling tools using on-machine laser tool setters. The remainder of this paper is organized as follows. Section 2 introduces the mechanism of flank wear formation in indexable face milling tools. Section 3 establishes the geometric model of the flank wear land width based on the insert and tool coordinate systems. Section 4 presents the flank wear land width measurement method using on-machine laser tool setters. Section 5 reports the experimental verification and industrial application of the proposed approach. Finally, Section 6 concludes the paper with the main findings.

## 2. Mechanism of Flank Wear Formation in Indexable Face Milling Tools

### 2.1. Overview of Indexable Face Milling Tools

Milling tools are primarily employed for machining slots and molding surfaces of various types and dimensions. These tools are typically designed as either solid tools or indexable insert tools. The latter offer significant versatility, as they can accommodate different types of inserts tailored to specific machining needs. Indexable face milling tools are extensively used in medium-scale to large-scale machining processes, especially for machining large planar workpieces due to their high cutting efficiency [44]. Their modular design permits the flexible combination of insert geometries, materials, and coatings to match a wide range of workpiece materials and cutting conditions. As such, they are highly suited for mass production environments.

**Figure 1:**
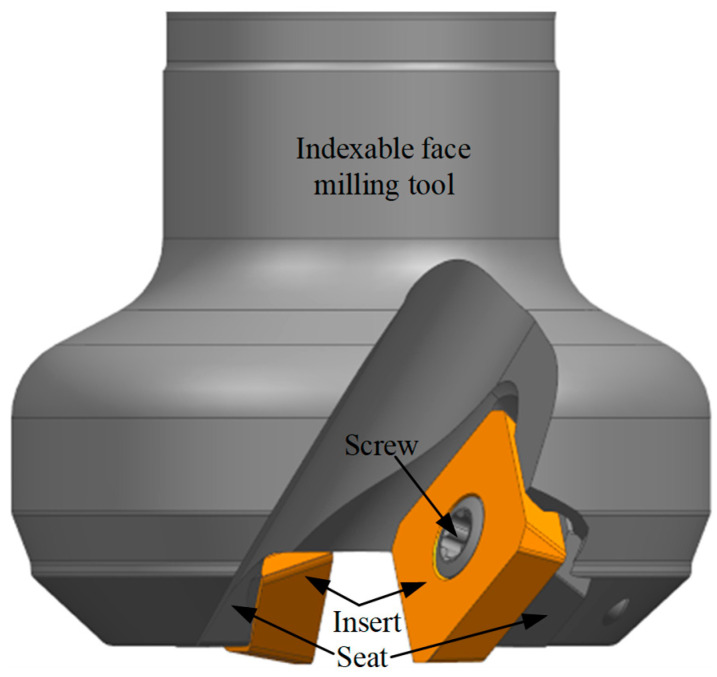
Schematic diagram of a typical indexable face milling tool.

A typical indexable face milling tool consists of one or more inserts, each matched in geometry and material to the workpiece being processed. As shown in Figure 1, the tool is mainly composed of inserts, screws, and a tool body with seats for the inserts. Each insert is mounted on the tool body seat using a screw and usually features one or more cutting edges. When the cutting edge of an insert becomes worn out or damaged, the screw is loosened to remove the insert, either rotating it to a new cutting edge or replacing it with a new insert. Finally, the screw is retightened to secure the insert in position, thereby maintaining overall tool integrity and machining precision. For indexable face milling tools, flank wear is the main form of tool wear. The tool condition is evaluated based on the width of the flank wear land. If the width exceeds a preset threshold, the insert is considered invalid, and the cutting edge should be indexed or the insert replaced.

### 2.2. Formation Process of the Flank Wear Land in Indexable Face Milling Tools

**Figure 2:**
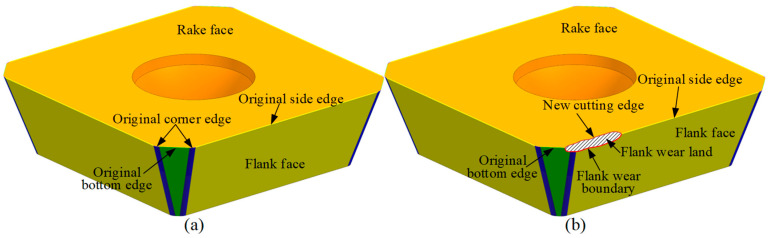
The components of insert. (**a**) A new insert and (**b**) a flank wear insert.

Since the tool flank face directly contacts the machined workpiece surface, it has a significant impact on the surface quality. Therefore, flank wear is typically used as a criterion for evaluating tool wear. As shown in Figure 2a, the cutting edge of the new indexable insert is composed of side edges, bottom edges, and corner edges. The side edge is located on the side of the tool and is mainly responsible for removing material from the side of the workpiece. The bottom edge is at the bottom of the tool and primarily removes material from the surface of the workpiece. The corner edge, usually located at the junction between the side and bottom edges, forms a rounded corner. During cutting, the corner edge serves to smooth the transition between the other two edges, thereby reducing cutting resistance and improving cutting efficiency. It also helps protect both the side and bottom edges from chipping, effectively extending the tool life.

As illustrated in Figure 2b, during the milling of a workpiece with an indexable face milling tool, the rotating tool causes the cutting edge of each insert to remove the workpiece material. At the same time, the flank face of the insert contacts the machined surface, generating friction and leading to flank wear. This results in the formation of an irregular flank wear land (the shaded area in the figure) and gradually creates a new cutting edge on the rake face (the black boundary line in the figure). As the machining continues, the area of the flank wear land gradually expands, eventually causing the tool failure. At this stage, the cutting must be stopped immediately and the cutting edge or insert replaced. Otherwise, the invalid tool will significantly reduce the machining quality of the workpiece or even cause it to be scrapped.

**Figure 3:**
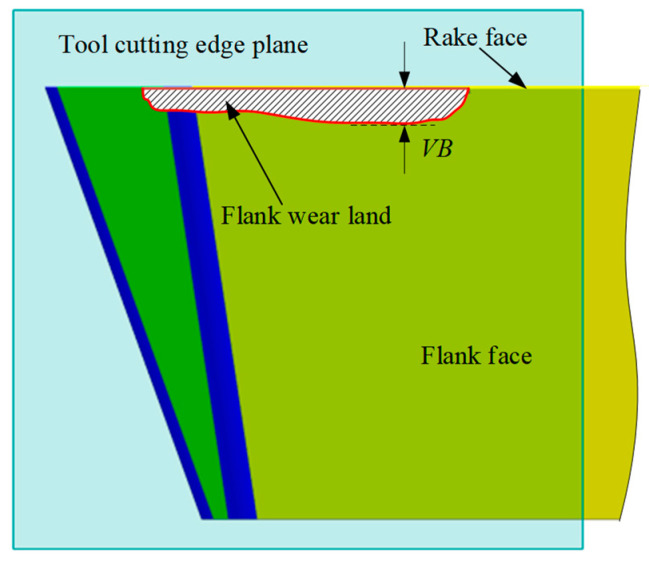
Illustration of the definition of the width of the flank wear land.

In the actual cutting process, the flank wear is affected by various factors, such as the geometry and materials of the tool and workpiece, as well as the machining parameters. Consequently, the width of the flank wear land is typically non-uniform. The width of the flank wear land is defined on a plane known as the tool cutting edge plane, which passes through the major cutting edge (the side edge of the insert) and is perpendicular to the rake face of the tool. The boundary of the flank wear land is projected onto this plane, and the perpendicular distance from each boundary projected point to the major cutting edge is defined as the width of the flank wear land (see Figure 3). In other words, the width of the flank wear land refers to the perpendicular distance from the wear boundary to the rake face. In this article, the maximum width is expressed as the flank wear value *VB*. The objective of tool condition monitoring (TCM) is to monitor the flank wear land and determine the maximum wear width in real-time, so that the tool can be changed before the wear value reaches or exceeds the preset threshold, thereby ensuring machining quality.

**Figure 4:**
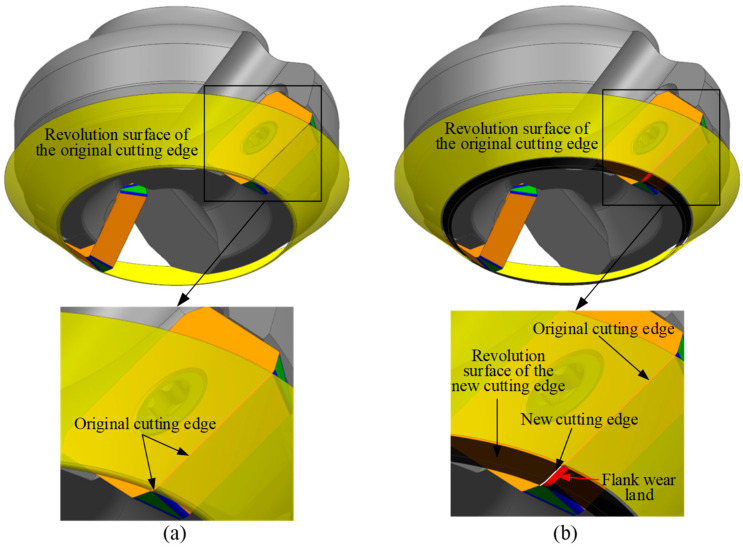
Schematic of the flank wear land formation process. (**a**) Revolution surface of the original cutting edge, and (**b**) revolution surface of the new cutting edge.

The formation process of the flank wear land in indexable face milling tools is as follows. During machining, the tool rotates around its axis, and the cutting edge of the insert generates a revolution surface (shown as the yellow surface in Figure 4a). The intersection between this revolving surface and the insert defines the original cutting edge. As the tool feeds into the workpiece, the part of the revolving surface within the axial depth of cut comes into contact with the workpiece and removes material. In the contact region, wear occurs on the cutting edge, forming a new cutting edge on the rake face (white curve in Figure 4b) and generating a flank wear land (red area in Figure 4b). As the tool continues to rotate, the new cutting edge generates a new revolution surface (see the black surface in Figure 4b), which further removes material from the workpiece. Consequently, the flank wear land gradually expands until its width reaches the preset threshold, leading to tool failure. It is important to note that the original cutting edge, which does not participate in the cutting process, still forms a revolution surface (see the yellow surface in Figure 4b). The flank wear land is a part of the revolution surface formed by the new cutting edge rotating around the tool axis, and its geometry is determined by the intersection between the new revolution surface and the worn insert. With the insert wear, the radius of the cutting edge gradually decreases. Therefore, a geometric relationship can be established between the width of the flank wear land at each point along the cutting edge and the tool radius reduction. By measuring the tool radius before and after wear using on-machine laser tool setters, the width of the flank wear land can be calculated and compared with the preset wear threshold to determine whether the tool is invalid, thereby achieving the objective of TCM.

## 3. Geometric Modeling of Flank Wear Land Width

Based on the above formation mechanism of the flank wear land in indexable face milling tools, a geometric relationship is established between the tool cutting edge radius and the width of the flank wear land. The purpose of this section is to derive a mathematical expression for the radius of the worn region at different axial heights to the corresponding flank wear land width, by analyzing the tool geometric model and the geometric characteristics of the wear region. A mathematical model is thereby established to describe the relationship between the tool radius and the flank wear land width.

### 3.1. Establishment of the Insert Coordinate System and Tool Coordinate System

**Figure 5:**
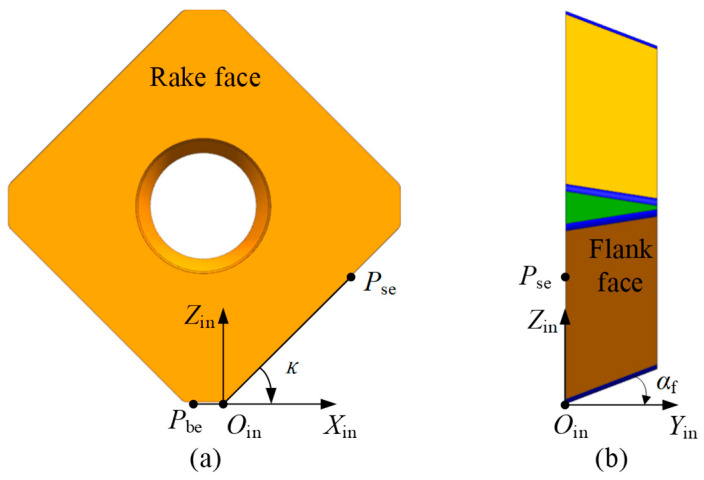
Illustration of the insert coordinate system. (**a**) Front view, and (**b**) side view.

In this paper, the focus is primarily on the cutting edge, rake face, and flank face of the insert. The geometric model of the insert is described in the insert coordinate system $X_{\mathrm{in}}Y_{\mathrm{in}}Z_{\mathrm{in}}O_{\mathrm{in}}$. Therefore, the $X_{\mathrm{in}}Y_{\mathrm{in}}Z_{\mathrm{in}}O_{\mathrm{in}}$ is first established. For an indexable insert with multiple cutting edges, the insert coordinate system is established as follows. As shown in Figure 5a, view the rake face of the insert from the front. A point $P_{\mathrm{se}}$ is selected on the right-side cutting edge, and a point $P_{\mathrm{be}}$ is selected on the adjacent bottom cutting edge. Draw a straight line $P_{\mathrm{se}}O_{\mathrm{in}}$ along the side edge at point $P_{\mathrm{se}}$, and another straight line $P_{\mathrm{be}}O_{\mathrm{in}}$ is drawn through $P_{\mathrm{be}}$ and extended along the bottom edge. The intersection of these two lines is a point $O_{\mathrm{in}}$, which is defined as a virtual cutting point determined during insert design. The origin of the insert coordinate system is defined at point $O_{\mathrm{in}}$. The $X_{\mathrm{in}}$ axis is defined along the vector direction of $\vec{P_{\mathrm{be}}O_{\mathrm{in}}}$; the $Z_{\mathrm{in}}$ axis passes through $O_{\mathrm{in}}$ and is perpendicular to the $X_{\mathrm{in}}$ axis, pointing from point $O_{\mathrm{in}}$ towards the inside of the insert as the positive direction of the $Z_{\mathrm{in}}$ axis. The $Y_{\mathrm{in}}$ axis is determined by the right-hand rule. According to the standard definitions of tool geometry, the angle between the vector $\vec{O_{\mathrm{in}}P_{\mathrm{se}}}$ and the $X_{\mathrm{in}}$ axis is defined as the entering angle of the insert, denoted as κ. The angle between the flank face of the insert and the $Y_{\mathrm{in}}$ axis is defined as the clearance angle, denoted as $\alpha_{f}$, as shown in Figure 5b.

**Figure 6:**
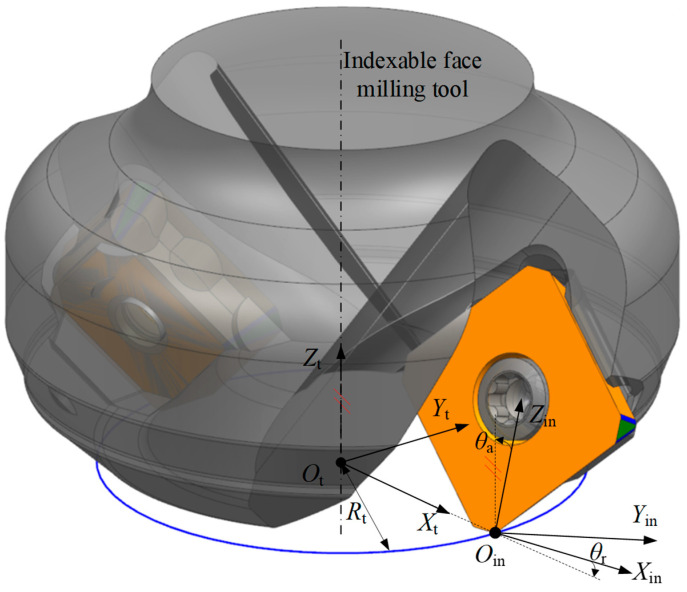
Illustration of the geometric relationship between the insert coordinate system and the tool coordinate system.

The insert of an indexable face milling tool is mounted into a seat on the tool body using screws. To describe the geometry of the insert within the tool, it is necessary to establish a tool coordinate system $X_{t}Y_{t}Z_{t}O_{t}$. As shown in Figure 6, the $Z_{t}$ axis is aligned with the geometric axis of the tool, i.e., the rotation axis of the tool holder. This ensures that the tool diameter, length, and flank wear measurements are accurately referenced along the true axial direction of the tool, avoiding misalignment errors. A plane perpendicular to the $Z_{t}$ axis is defined to pass through the origin $O_{\mathrm{in}}$, and this plane is referred to as the tool end plane. The intersection point between the tool end face and the $Z_{t}$ axis is defined as the origin $O_{t}$. The $X_{t}$ axis is defined along the vector from $O_{t}$ to $O_{\mathrm{in}}$, and the $Y_{t}$ axis is determined according to the right-hand rule. It is worth noting that both the $X_{t}$ and $X_{\mathrm{in}}$ axes lie in the $X_{t}O_{t}Y_{t}$ plane.

According to the design of the indexable face milling tool, when the insert is mounted into the insert seat, the inclination of the insert cutting face is represented by the rake angle. There are two types of rake angles: the axial rake angle $\theta_{a}$ and the radial rake angle $\theta_{r}$. The axial rake angle $\theta_{a}$ is the angle between the rake face of the insert and the tool axis $Z_{t}$, while the radial rake angle $\theta_{r}$ is the angle between the $X_{\mathrm{in}}$ and $X_{t}$ axes. The distance from $O_{t}$ to $O_{\mathrm{in}}$ is typically defined as the tool radius, denoted as $R_{t}$. To transform the insert coordinate system $X_{\mathrm{in}}Y_{\mathrm{in}}Z_{\mathrm{in}}O_{\mathrm{in}}$ into the tool coordinate system $X_{t}Y_{t}Z_{t}O_{t}$, the following steps are performed. First, rotate the $X_{\mathrm{in}}Y_{\mathrm{in}}Z_{\mathrm{in}}O_{\mathrm{in}}$ about the $X_{\mathrm{in}}$ axis by the axial rake angle $\theta_{a}$. Then, rotate about the $Z_{\mathrm{in}}$ axis by the radial rake angle $\theta_{r}$. Finally, translate along the direction from $O_{\mathrm{in}}$ to $O_{t}$ by a distance of $R_{t}$. The resulting coordinate system is defined as the tool coordinate system $X_{t}Y_{t}Z_{t}O_{t}$.

According to the above geometric relationship in Figure 6, the transformation relationship between the insert coordinate system $X_{\mathrm{in}}Y_{\mathrm{in}}Z_{\mathrm{in}}O_{\mathrm{in}}$ and the tool coordinate system $X_{t}Y_{t}Z_{t}O_{t}$ can be given by the following homogeneous transformation matrix.

(1) $$M_{\mathrm{in}}^{t}=\left[\begin{array}{cccc} \cos\theta_{r} & \sin\theta_{r}\cos\theta_{a} & \sin\theta_{r}\sin\theta_{a} & R_{t}\\ -\sin\theta_{r} & \cos\theta_{r}\cos\theta_{a} & \cos\theta_{r}\sin\theta_{a} & 0\\ 0 & -\sin\theta_{a} & \cos\theta_{a} & 0\\ 0 & 0 & 0 & 1 \end{array}\right]$$

### 3.2. Geometric Model of the Indexable Face Milling Tool and Flank Wear Land Width

**Figure 7:**
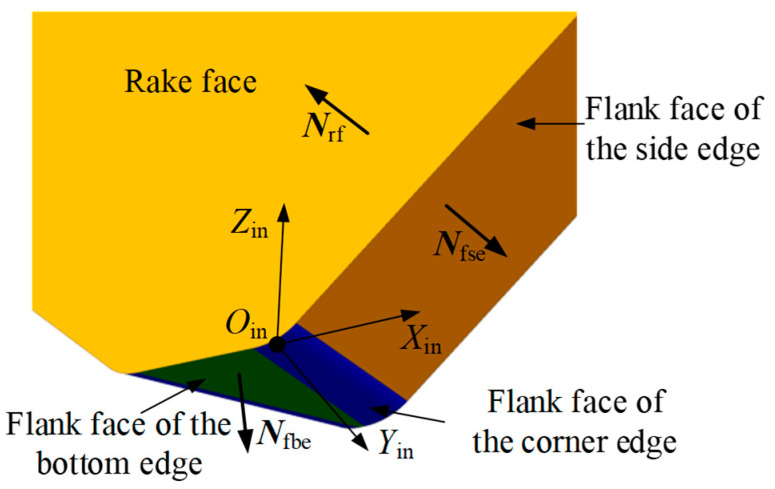
Illustration of the unit normal vectors of the rake face and the flank face.

Extensive experiments have shown that during the machining of large flat surfaces with indexable face milling tools, wear mainly occurs on the side edge and the corner edge. Therefore, this study primarily focuses on the flank wear land in these two regions. According to the classification of cutting edges, the flank face is divided into the flank face of the side edge, the flank face of the corner edge, and the flank face of the bottom edge. As shown in Figure 7, based on the geometric model of the insert, the normal vector expressions of the rake face and the flank face are derived in the insert coordinate system.

The rake face does not vary with the type of cutting edge; therefore, a single insert has only one rake face. The expression for its unit normal vector in the insert coordinate system $X_{\mathrm{in}}Y_{\mathrm{in}}Z_{\mathrm{in}}O_{\mathrm{in}}$ is

(2) $$N_{\mathrm{rf}}^{\mathrm{in}}={\left[\begin{array}{cccc} 0 & -1 & 0 & 0 \end{array}\right]}^{T}$$

The expression for the unit normal vector of the side edge flank face in the insert coordinate system $X_{\mathrm{in}}Y_{\mathrm{in}}Z_{\mathrm{in}}O_{\mathrm{in}}$ is

(3) $$N_{\mathrm{fse}}^{\mathrm{in}}={\left[\begin{array}{cccc} \sin\kappa \cos\alpha_{f} & \sin\alpha_{f} & -\cos\kappa \cos\alpha_{f} & 0 \end{array}\right]}^{T}$$

The expression for the unit normal vector of the bottom edge flank face in the insert coordinate system $X_{\mathrm{in}}Y_{\mathrm{in}}Z_{\mathrm{in}}O_{\mathrm{in}}$ is

(4) $$N_{\mathrm{fbe}}^{\mathrm{in}}={\left[\begin{array}{cccc} 0 & \sin\alpha_{f} & -\cos\alpha_{f} & 0 \end{array}\right]}^{T}$$

Based on the definition of the width of the tool flank wear land, the wear land width is the perpendicular distance from a point on the boundary of the wear land to the rake face. Therefore, it is necessary to derive the parametric equation of the rake face. Based on Equations (1) and (2), the unit normal vector of the rake face in the tool coordinate system $X_{t}Y_{t}Z_{t}O_{t}$ is expressed as

(5) $$N_{\mathrm{rf}}^{t}=\left[\begin{array}{c} N_{\mathrm{rf},x}^{t}\\ N_{\mathrm{rf},y}^{t}\\ N_{\mathrm{rf},z}^{t}\\ 0 \end{array}\right]=M_{\mathrm{in}}^{t}\cdot N_{\mathrm{rf}}^{\mathrm{in}}=\left[\begin{array}{c} -\sin\theta_{r}\cos\theta_{a}\\ -\cos\theta_{r}\cos\theta_{a}\\ \sin\theta_{a}\\ 0 \end{array}\right]$$

From the establishment process of the insert coordinate system and the tool coordinate system, it is known that the intersection point $O_{\mathrm{in}}$ lies on the plane where the tool rake face is located. Its coordinates in the tool coordinate system are $O_{\mathrm{in}}^{t}={\left[\begin{array}{cccc} R_{t} & 0 & 0 & 1 \end{array}\right]}^{T}$. Therefore, the equation of the rake face in the tool coordinate system $X_{t}Y_{t}Z_{t}O_{t}$ can be written as

(6) $$N_{\mathrm{rf},x}^{t}\left(x-R_{t}\right)+N_{\mathrm{rf},y}^{t}y+N_{\mathrm{rf},z}^{t}z=0$$

#### 3.2.1. Flank Wear Land Width of the Side Edge

**Figure 8:**
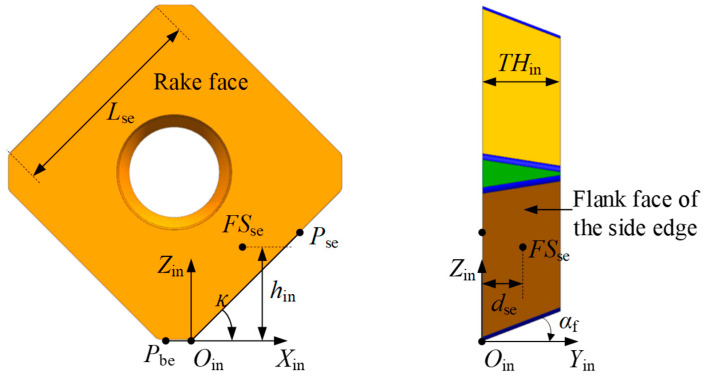
Illustration of the geometric model of the flank face of the side edge.

When the axial depth of cut is relatively large, the side edge comes into contact with the workpiece material, resulting in wear on the side edge. The following section derives the geometrical model of the side edge flank face and the corresponding parametric equations of the flank wear land width. As shown in Figure 8, a point $FS_{\mathrm{se}}$ is selected on the flank face of the side edge. Based on the geometric relationships illustrated in the figure, the flank face of the side edge in the insert coordinate system $X_{\mathrm{in}}Y_{\mathrm{in}}Z_{\mathrm{in}}O_{\mathrm{in}}$ can be described by the following parametric equation.

(7) $$FS_{\mathrm{se}}^{\mathrm{in}}=\left[\begin{array}{c} FS_{\mathrm{se},x}^{\mathrm{in}}\\ FS_{\mathrm{se},y}^{\mathrm{in}}\\ FS_{\mathrm{se},z}^{\mathrm{in}}\\ 1 \end{array}\right]=\left[\begin{array}{c} h_{\mathrm{in}}\tan\kappa -\frac{d_{\mathrm{se}}\tan\alpha_{f}}{\sin\kappa }\\ d_{\mathrm{se}}\\ h_{\mathrm{in}}\\ 1 \end{array}\right]$$

where $h_{\mathrm{in}}$ and $d_{\mathrm{se}}$ are variable parameters, $h_{\mathrm{in}}\in \left[r_{c}\left(1-\cos\kappa \right),r_{c}\left(1-\cos\kappa \right)+L_{\mathrm{se}}\sin\kappa \right]$, $d_{\mathrm{se}}\in \left[0,TH_{\mathrm{in}}\right]$; $TH_{\mathrm{in}}$ is the thickness of the insert, $r_{c}$ is the radius of the corner edge, and $L_{\mathrm{se}}$ is the length of the side edge.

Combining with Equation (1), the parametric equation of the flank face of the side edge in the tool coordinate system $X_{t}Y_{t}Z_{t}O_{t}$ is derived as follows.

(8) $$FS_{\mathrm{se}}^{t}=\left[\begin{array}{c} FS_{\mathrm{se},x}^{t}\\ FS_{\mathrm{se},y}^{t}\\ FS_{\mathrm{se},z}^{t}\\ 1 \end{array}\right]=M_{\mathrm{in}}^{t}\cdot FS_{\mathrm{se}}^{\mathrm{in}}=\left[\begin{array}{c} \left(h_{\mathrm{in}}\tan\kappa -\frac{d_{\mathrm{se}}\tan\alpha_{f}}{\sin\kappa }\right)\cos\theta_{r}+\left(d_{\mathrm{se}}\cos\theta_{a}+h_{\mathrm{in}}\sin\theta_{a}\right)\sin\theta_{r}+R_{t}\\ -\left(h_{\mathrm{in}}\tan\kappa -\frac{d_{\mathrm{se}}\tan\alpha_{f}}{\sin\kappa }\right)\sin\theta_{r}+\left(d_{\mathrm{se}}\cos\theta_{a}+h_{\mathrm{in}}\sin\theta_{a}\right)\cos\theta_{r}\\ -d_{\mathrm{se}}\sin\theta_{a}+h_{\mathrm{in}}\cos\theta_{a}\\ 1 \end{array}\right]$$

**Figure 9:**
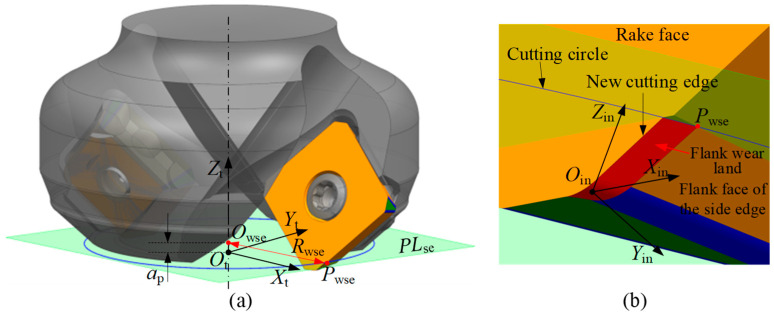
Schematic diagram of the cutting circle and wear point on the flank face of the side edge. (**a**) Global view and (**b**) local enlarged view of the wear region.

In the milling process, the cutting parameters are defined with reference to the tool coordinate system. Assuming that the axial depth of cut is $a_{p}$, then the cutting edge located within the range of distance $a_{p}$ from the tool end plane may experience flank wear, forming a flank wear land (see the red area in Figure 9b), and generating a new cutting edge. As shown in Figure 9a, a plane is constructed parallel to the $X_{t}O_{t}Y_{t}$ plane and located at a distance $a_{p}$ from it. This plane is referred to as the side edge flank wear plane $PL_{\mathrm{se}}$. The intersection of this plane with the boundary of the flank wear land defines a point, which is referred to as the side edge wear point $P_{\mathrm{wse}}$. As cutting continues, this point will form a new cutting circle, which constitutes part of the revolution surface of the cutting edge. On the side edge flank wear plane $PL_{\mathrm{se}}$, a circle is drawn through the side edge wear point $P_{\mathrm{wse}}$ with radius equal to its distance from the tool axis. This circle is called the cutting circle, and its radius is denoted as $R_{\mathrm{wse}}$. According to the geometric relationships shown in Figure 9, the coordinates of the point $P_{\mathrm{wse}}$ in the tool coordinate system can be calculated.

The side edge wear point $P_{\mathrm{wse}}$ lies on the flank face of the side edge of the tool; therefore, the coordinates of point $P_{\mathrm{wse}}$ satisfy Equation (8). Based on the axial depth of cut, the z-coordinate of point $P_{\mathrm{wse}}$ is $P_{\mathrm{wse},z}^{t}=a_{p}$. Additionally, since point $P_{\mathrm{wse}}$ lies on the cutting circle, its distance to the tool axis equals the radius $R_{\mathrm{wse}}$. By combining the above conditions, the coordinates of the wear point $R_{\mathrm{wse}}$ in the tool coordinate system $X_{t}Y_{t}Z_{t}O_{t}$ can be determined using Equation (9).

(9) $$\left\{\begin{array}{l} P_{\mathrm{wse},x}^{t}=\cos\theta_{r}\left(h_{\mathrm{in}}\tan\kappa -\frac{d_{\mathrm{se}}\tan\alpha_{f}}{\sin\kappa }\right)+\sin\theta_{r}\left(d_{\mathrm{se}}\cos\theta_{a}+h_{\mathrm{in}}\sin\theta_{a}\right)+R_{t}\\ P_{\mathrm{wse},y}^{t}=-\sin\theta_{r}\left(h_{\mathrm{in}}\tan\kappa -\frac{d_{\mathrm{se}}\tan\alpha_{f}}{\sin\kappa }\right)+\cos\theta_{r}\left(d_{\mathrm{se}}\cos\theta_{a}+h_{\mathrm{in}}\sin\theta_{a}\right)\\ P_{\mathrm{wse},z}^{t}=-d_{\mathrm{se}}\sin\theta_{a}+h_{\mathrm{in}}\cos\theta_{a}\\ P_{\mathrm{wse},z}^{t}=a_{p}\\ {P_{\mathrm{wse},x}^{t}}^{2}+{P_{\mathrm{wse},y}^{t}}^{2}={R_{\mathrm{wse}}}^{2} \end{array}\right.$$

where $P_{\mathrm{wse},x}^{t}$, $P_{\mathrm{wse},y}^{t}$, and $P_{\mathrm{wse},z}^{t}$ represent the coordinate values of the wear point $P_{\mathrm{wse}}$ in the tool coordinate system, which can be obtained by solving Equation (9), and $a_{p}\geq r_{c}\left(1-\cos\kappa \right)\cos\theta_{a}$.

It is important to note that in the derivation process, only the measurement height is set as the axial depth of cut $a_{p}$, and the corresponding coordinates of the wear point $P_{\mathrm{wse}}$ are calculated. For wear points at other heights within the wear land, the coordinates can be obtained by adjusting the measurement height and the corresponding radius in Equation (9), thereby providing data support for calculating the flank wear width.

According to the definition of the width of the tool flank wear land, this width is the perpendicular distance from the wear point $P_{\mathrm{wse}}$ to the rake face. Therefore, by combining the plane equation of the rake face (see Equation (6)) and substituting the coordinate values obtained from Equation (9) into Equation (10), the width of the wear land at point $P_{\mathrm{wse}}$ can be calculated.

(10) $$VB_{\mathrm{wse}}=\left|-\left(P_{\mathrm{wse},x}^{t}-R_{t}\right)\sin\theta_{r}\cos\theta_{a}-P_{\mathrm{wse},y}^{t}\cos\theta_{r}\cos\theta_{a}+P_{\mathrm{wse},z}^{t}\sin\theta_{a}\right|$$

#### 3.2.2. Flank Wear Land Width of the Corner Edge

When the axial depth of cut is small, it is possible that only the corner edge comes into contact with the workpiece material, resulting in wear on the corner edge. The following section derives the geometric model of the flank face of the corner edge and the corresponding parametric equation for the flank wear land width.

**Figure 10:**
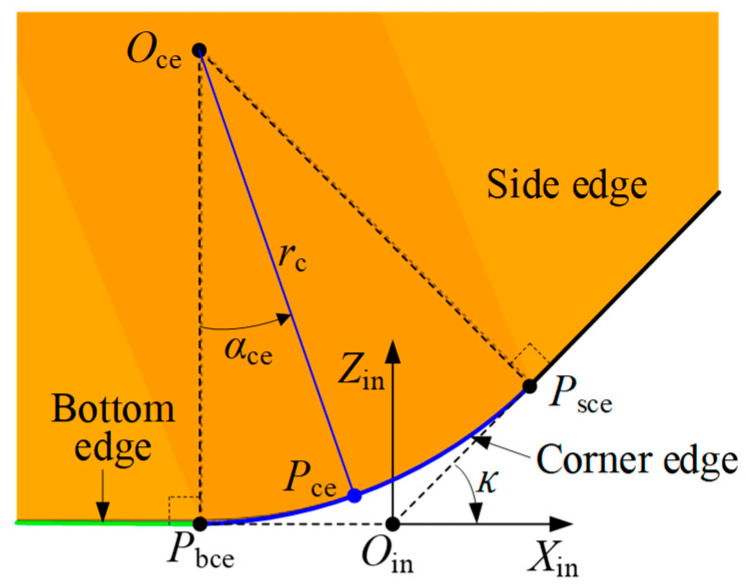
Illustration of the geometric model of the corner side edge.

Figure 10 illustrates the geometric model of the corner edge. In the figure, the green line represents the bottom edge, the black line represents the side edge, and the blue curve represents the corner edge. From a geometrical perspective, the corner edge is tangent to the bottom edge and the side edge at points $P_{\mathrm{bce}}$ and $P_{\mathrm{sce}}$, respectively. By selecting a point $P_{\mathrm{ce}}$ on the corner edge, the parametric equation of the corner edge in the insert coordinate system $X_{\mathrm{in}}Y_{\mathrm{in}}Z_{\mathrm{in}}O_{\mathrm{in}}$ can be expressed as follows based on the geometric relationships shown in Figure 10.

(11) $$P_{\mathrm{ce}}^{\mathrm{in}}=\left[\begin{array}{c} P_{\mathrm{ce},x}^{\mathrm{in}}\\ P_{\mathrm{ce},y}^{\mathrm{in}}\\ P_{\mathrm{ce},z}^{\mathrm{in}}\\ 1 \end{array}\right]=\left[\begin{array}{c} r_{c}\left(\sin\alpha_{\mathrm{ce}}-\tan\left(\kappa /2\right)\right)\\ 0\\ r_{c}\left(1-\cos\alpha_{\mathrm{ce}}\right)\\ 1 \end{array}\right]$$

where $r_{c}$ is the radius of the corner edge; $\alpha_{\mathrm{ce}}$ is a variable parameter, and $\alpha_{\mathrm{ce}}\in \left(0,\kappa \right)$; κ is the entering angle of the insert.

**Figure 11:**
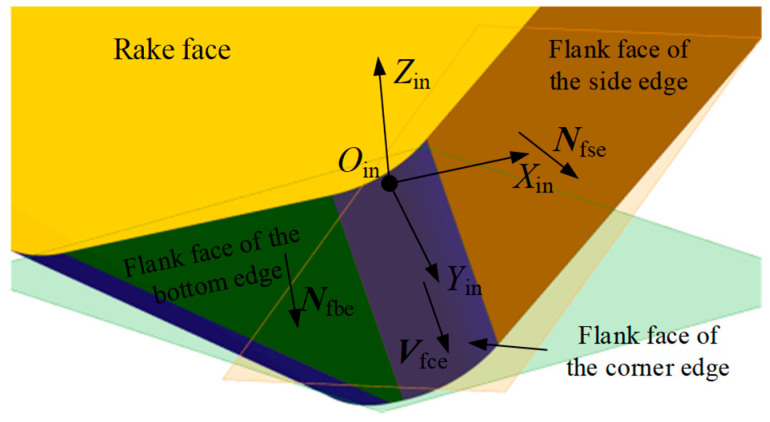
Illustration of the geometric model of the flank face of the corner edge.

The flank face of the corner edge lies between the flank face of the side edge and the bottom edge, and is a part of the cylindrical side surface, as shown in Figure 11. Geometrically, the flank face of the corner edge can be generated by sweeping the corner edge along a direction $V_{\mathrm{fce}}$, where this direction corresponds to the intersection line between the flank face of the side edge (see the translucent orange plane in the Figure 11) and the bottom edge (see the translucent green plane in the Figure 11).

Based on the unit normal vector formulas of the flank face of the side edge (Equation (3)) and the flank face of the bottom edge (Equation (4)), the sweeping direction $V_{\mathrm{fce}}$ can be calculated by the following equation.

(12) $$V_{\mathrm{fce}}=N_{\mathrm{fse}}^{\mathrm{in}}\times N_{\mathrm{fbe}}^{\mathrm{in}}=\left[\begin{array}{c} \sin\alpha_{f}\cos\alpha_{f}\left(\cos\kappa -1\right)\\ \sin\kappa \cos{\alpha_{f}}^{2}\\ \sin\kappa \sin\alpha_{f}\cos\alpha_{f}\\ 0 \end{array}\right]$$

By combining Equation (11), the parametric equation of the flank face of the corner edge in the insert coordinate system $X_{\mathrm{in}}Y_{\mathrm{in}}Z_{\mathrm{in}}O_{\mathrm{in}}$ can be expressed as follows.

(13) $$FS_{\mathrm{ce}}^{\mathrm{in}}=\left[\begin{array}{c} FS_{\mathrm{ce},x}^{\mathrm{in}}\\ FS_{\mathrm{ce},y}^{\mathrm{in}}\\ FS_{\mathrm{ce},z}^{\mathrm{in}}\\ 1 \end{array}\right]=P_{\mathrm{ce}}^{\mathrm{in}}+l_{\mathrm{ce}}\cdot V_{\mathrm{fce}}=\left[\begin{array}{c} r_{c}\left(\sin\alpha_{\mathrm{ce}}-\tan\left(\kappa /2\right)\right)+l_{\mathrm{ce}}\sin\alpha_{f}\cos\alpha_{f}\left(\cos\kappa -1\right)\\ l_{\mathrm{ce}}\sin\kappa \cos{\alpha_{f}}^{2}\\ r_{c}\left(1-\cos\alpha_{\mathrm{ce}}\right)+l_{\mathrm{ce}}\sin\kappa \sin\alpha_{f}\cos\alpha_{f}\\ 1 \end{array}\right]$$

where $\alpha_{\mathrm{ce}}$ and $l_{\mathrm{ce}}$ are variable parameters, $\alpha_{\mathrm{ce}}\in \left(0,\kappa \right)$ and $l_{\mathrm{ce}}\in \left[0,TH_{\mathrm{in}}/\cos\alpha_{f}\right]$.

Thus, combining Equation (1), the parametric equation of the flank face of the corner edge in the tool coordinate system $X_{t}Y_{t}Z_{t}O_{t}$ is derived as follows.

(14) $$FS_{\mathrm{ce}}^{t}=\left[\begin{array}{c} FS_{\mathrm{ce},x}^{t}\\ FS_{\mathrm{ce},y}^{t}\\ FS_{\mathrm{ce},z}^{t}\\ 1 \end{array}\right]=M_{\mathrm{in}}^{t}\cdot FS_{\mathrm{ce}}^{\mathrm{in}}=\left[\begin{array}{c} FS_{\mathrm{ce},x}^{\mathrm{in}}\cos\theta_{r}+\left(FS_{\mathrm{ce},y}^{\mathrm{in}}\cos\theta_{a}+FS_{\mathrm{ce},z}^{\mathrm{in}}\sin\theta_{a}\right)\sin\theta_{r}+R_{t}\\ -FS_{\mathrm{ce},x}^{\mathrm{in}}\sin\theta_{r}+\left(FS_{\mathrm{ce},y}^{\mathrm{in}}\cos\theta_{a}+FS_{\mathrm{ce},z}^{\mathrm{in}}\sin\theta_{a}\right)\cos\theta_{r}\\ -FS_{\mathrm{ce},y}^{\mathrm{in}}\sin\theta_{a}+FS_{\mathrm{ce},z}^{\mathrm{in}}\cos\theta_{a}\\ 1 \end{array}\right]$$

**Figure 12:**
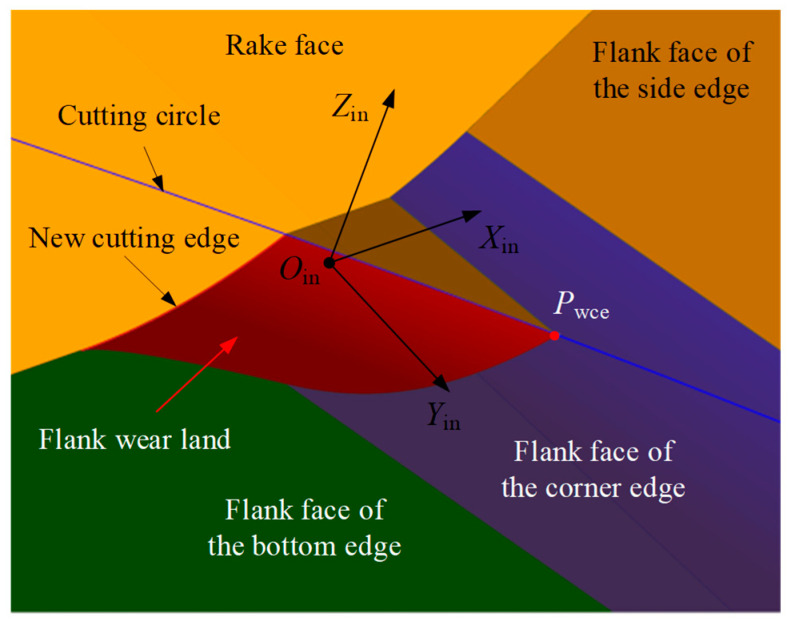
Illustration of the cutting circle and wear point on the flank face of the corner edge.

Similarly to the flank wear land of the side edge, when the axial depth of cut is $a_{p}$, the cutting edges within the range $a_{p}$ from the tool end plane will undergo flank face wear, forming the flank wear land (the red region shown in Figure 12), and generating a new cutting edge. The difference is that when the axial depth of cut $a_{p}$ is small, wear only occurs on the flank face of the corner edge. Similarly, the wear plane of the flank face of the corner edge intersects the boundary of the flank wear land at the corner edge wear point $P_{\mathrm{wce}}$. During continued cutting, this point generates a new cutting circle, which forms part of the revolution surface of the cutting edge. The radius of the cutting circle on the flank face of the corner edge is denoted as $R_{\mathrm{wce}}$. Based on the geometric relationships in Figure 12, the coordinates of the wear point $P_{\mathrm{wce}}$ in the tool coordinate system can be calculated.

The corner edge wear point $P_{\mathrm{wce}}$ is located on the flank face of the corner edge of the tool; thus, the coordinates of point $P_{\mathrm{wce}}$ satisfy Equation (14). Additionally, according to the axial depth of cut $a_{p}$ and the radius $R_{\mathrm{wce}}$ of the cutting circle, the coordinates of the wear point $P_{\mathrm{wce}}$ in the tool coordinate system $X_{t}Y_{t}Z_{t}O_{t}$ can be determined using Equation (15).

(15) $$\left\{\begin{array}{l} P_{\mathrm{wce},x}^{t}=FS_{\mathrm{ce},x}^{\mathrm{in}}\cos\theta_{r}+\left(FS_{\mathrm{ce},y}^{\mathrm{in}}\cos\theta_{a}+FS_{\mathrm{ce},z}^{\mathrm{in}}\sin\theta_{a}\right)\sin\theta_{r}+R_{t}\\ P_{\mathrm{wce},y}^{t}=-FS_{\mathrm{ce},x}^{\mathrm{in}}\sin\theta_{r}+\left(FS_{\mathrm{ce},y}^{\mathrm{in}}\cos\theta_{a}+FS_{\mathrm{ce},z}^{\mathrm{in}}\sin\theta_{a}\right)\cos\theta_{r}\\ P_{\mathrm{wce},z}^{t}=-FS_{\mathrm{ce},y}^{\mathrm{in}}\sin\theta_{a}+FS_{\mathrm{ce},z}^{\mathrm{in}}\cos\theta_{a}\\ P_{\mathrm{wce},z}^{t}=a_{p}\\ {P_{\mathrm{wce},x}^{t}}^{2}+{P_{\mathrm{wce},y}^{t}}^{2}={R_{\mathrm{wce}}}^{2} \end{array}\right.$$

where $P_{\mathrm{wce},x}^{t}$, $P_{\mathrm{wce},y}^{t}$, and $P_{\mathrm{wce},z}^{t}$ represent the coordinate values of the wear point $P_{\mathrm{wce}}$ in the $X_{t}Y_{t}Z_{t}O_{t}$, which can be obtained by solving Equation (15), and $a_{p}\in \left(0,r_{c}\left(1-\cos\kappa \right)\cos\theta_{a}\right)$.

By the same reasoning, substituting the coordinate values obtained from Equation (15) into Equation (16), the wear land width at the corner edge wear point $P_{\mathrm{wce}}$ can be calculated.

(16) $$VB_{\mathrm{wce}}=\left|-\left(P_{\mathrm{wce},x}^{t}-R_{t}\right)\sin\theta_{r}\cos\theta_{a}-P_{\mathrm{wce},y}^{t}\cos\theta_{r}\cos\theta_{a}+P_{\mathrm{wce},z}^{t}\sin\theta_{a}\right|$$

In summary, based on the tool measurement height and the corresponding tool radius value, the flank wear width of the corresponding wear point can be calculated using either Equation (10) or Equation (16).

## 4. Flank Wear Land Width Measurement Method Based on Laser Tool Setters

This core purpose of this study is to achieve real-time compensation and condition monitoring of cutting tools during machining by utilizing on-machine tool measurement systems. Specifically, flank wear width is used as the primary indicator for assessing tool condition. To this end, a novel monitoring approach based on the laser tool setter is proposed, comprising two main tasks.

First, the focus is on monitoring tool size variations throughout the machining process using the laser tool setter, while simultaneously acquiring tool wear images and measuring wear values with a tool microscope, to identify wear patterns and determine the critical wear threshold. In actual machining environments, due to errors in tool manufacturing and installation, as well as variations in material properties and unreasonable cutting parameters, flank wear on the cutting edge often displays non-uniform characteristics. The wear amount of the cutting edge varies greatly at different heights, with some positions along the axial direction exhibit significantly greater wear than others. It is known that these severely worn areas tend to degrade more rapidly, accelerating the overall deterioration of the tool performance and leading to premature failure. The laser tool setter, known for its high precision and on-machine measurement capability, is widely adopted in CNC machining. It enables efficient measurement of tool radius at specific axial positions. In theory, the cutting edge within the range of the axial depth of cut experiences wear. By measuring the tool radius at each height within this range, a comprehensive profile of the tool wear can be obtained, enabling accurate determination of the tool wear condition. However, measuring the tool radius at all heights would significantly increase machining time and production costs, making this approach not feasible in practice. Therefore, it is essential to optimize the measurement positions along the tool axis to balance accuracy in wear evaluation with measurement efficiency. Moreover, the wear status and wear threshold are often influenced by multiple factors and thus cannot be assumed fixed. To determine the optimal heights for radius measurement and the corresponding wear threshold, dedicated cutting experiments are carried out. In these experiments, actual cutting tools and workpiece materials are employed. The tool radius measurements are conducted at various heights at regular intervals. Simultaneously, wear images are collected using tool microscope until tool failure occurs. By analyzing these images of tool wear conditions, the dominant wear pattern of the tool is identified. Particular attention is given to the quantitative analysis of the flank wear land width, aiming to reveal the spatial distribution characteristics of flank wear, to optimize the height of the tool radius measurement. Meanwhile, the calculated flank wear land widths are obtained using the calculation method proposed in Section 3, which is compared with the measurement results to verify the accuracy of the calculation method. Based on the experimental results, the dominant wear pattern is summarized, and the axial heights with the largest flank wear and leading to tool failure were selected as the optimized height of the tool radius measurement. The flank wear land width at tool failure is then used to define a wear threshold, which serves as a criterion for real-time TCM.

Second, with the optimized height identified, the laser tool setter is used to measure the radius at that specific axial location and update the tool dimension in the machine controller’s tool offset list. Specifically, the tool radius is measured at the optimized height, and the corresponding flank wear land width is calculated using the method proposed in Section 3. The wear widths obtained at all measured heights are comprehensively evaluated and compared with the predefined wear threshold. If all calculated wear widths are below the threshold, the tool is considered to be in valid condition. However, if any calculated width exceeds the threshold, the tool is judged to be invalid, indicating that the insert or cutting edge needs to be replaced.

## 5. Experimental Verification and Application

To verify the accuracy of the proposed flank wear land width calculation method and evaluate the practicality of the TCM approach presented in this study, a series of cutting experiments were conducted under representative conditions that reflect typical milling operations. The experiments are divided into two parts: The objective of Experiment I is to identify the wear pattern of the experimental tools, optimize the height of the tool radius measurement, validate the accuracy of the flank wear land width calculation method, and determine the corresponding wear threshold. Experiment II is an application-oriented test designed to assess the feasibility and effectiveness of the TCM method developed based on the results of Experiment I.

**Figure 13:**
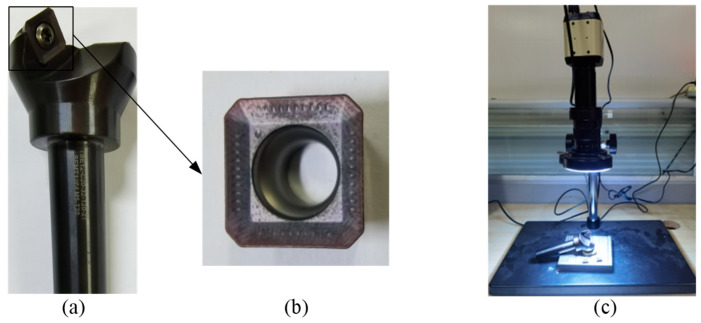
Main equipment used in the experiments. (**a**) An indexable face milling tool; (**b**) an insert and (**c**) a tool microscope.

All experiments were conducted on a four-axis vertical machining center, model YHVT850Z, and its manufacturer is Shandong Yonghua Machinery Co., Ltd. in Jining, China. The machining center is equipped with a Siemens 840D sl CNC system and a BT40 spindle tool holder. The non-contact laser tool setter used in the experiments was the NC4 system from Renishaw, Wales, UK, specifically the modular fixed model F300. The indexable face milling tool used in the experiment is model TSE12-C20-32-120-2T, with an overall shank length of 120 mm, a shank diameter of 20 mm, and a nominal tool diameter of 32 mm. This milling tool is equipped with two square inserts. The insert model is SEKT1204, made of cemented carbide with a TiAlN coating. The manufacturer of the indexable face milling tool and insert is Dongguan Yiyang Precision Hardware Co., Ltd. in Dongguan, China. Detailed specifications of the indexable face milling tool and inserts are listed in Table 1. The workpiece material is 2Cr13 stainless steel with a hardness of HRC 20 and dimensions of 110 mm × 110 mm × 35 mm. The manufacturer is Jiangsu Hengshuntai Steel Co., Ltd., Wuxi, China. Meanwhile, the tool wear condition images were captured and measured via the YW2300 tool microscope manufactured by Shenzhen Yangwang Technology Co., Ltd., Shenzhen, China, and the device evaluates the flank wear width (*VB*), which is used as the reference value for the experiments. The main equipment used in the experiment is shown in Figure 13.

### 5.1. Experiment I

Through cutting experiments, wear images of the tool at different machining stages were captured using a tool microscope to identify the primary wear pattern. The flank wear data were measured to study the distribution characteristics of the wear land and determine the position of maximum wear. The tool radius at different heights was measured using a laser tool setter, and the flank wear land width was calculated. By comparing the calculated wear land results with the measured values to verify the accuracy of the wear land width calculation model. Based on the experimental results, a threshold for wear band width was established to provide a quantitative basis for TCM.

**Figure 14:**
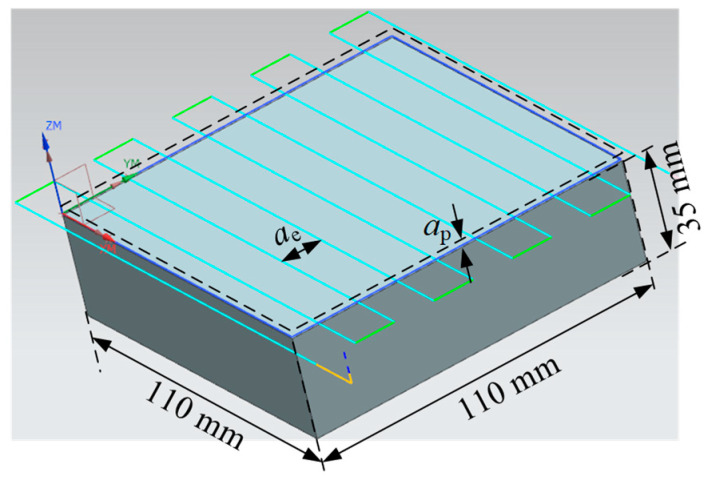
Reciprocating cutting tool path.

The cutting experiments were conducted using face milling to machine stainless steel blocks, with a reciprocating milling strategy (see Figure 14) under dry cutting conditions. Based on the tool manufacturer’s catalog, the selected cutting parameters are listed in Table 2. In order to eliminate surface irregularities of the workpiece and ensure consistent cutting depth during the experiment, a preprocessing step was performed before the cutting experiment by machining a depth of 0.5 mm from the workpiece surface.

**Figure 15:**
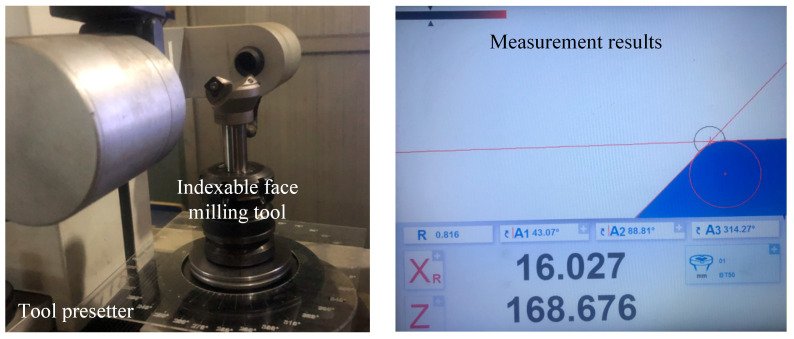
Schematic diagram of adjusting tool runout and measuring the actual tool radius using a tool presetter.

Due to manufacturing and installation errors of the indexable face milling tool and its inserts, the actual tool radius of the insert mounted on the milling tool may deviate from the nominal radius. Therefore, before the cutting experiments, the actual tool radius was measured using an offline tool presetter. The inserts were then manually adjusted to ensure that both axial and radial runout remained within 0.005 mm, in order to minimize the influence of tool length and radius variations on the experimental results, as shown in Figure 15.

**Figure 16:**
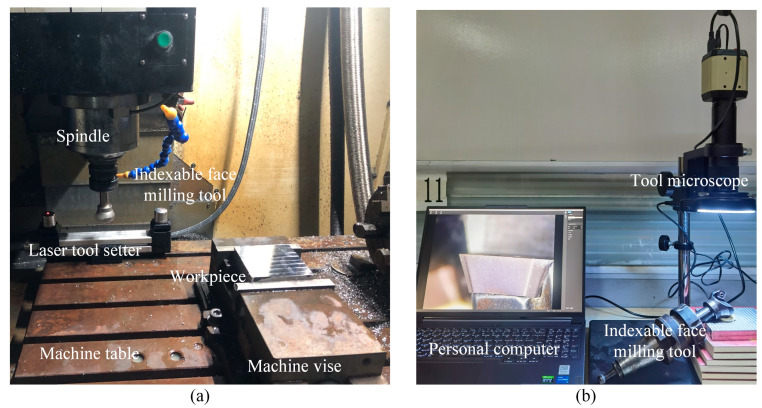
Actual cutting experiment setup. (**a**) Milling process and tool dimension measurement, and (**b**) capturing and measuring tool wear.

During the entire cutting experiment, the tool length and radius were measured using a laser tool setter. The tool was removed from the machine spindle to capture images of the insert wear condition and to measure the tool wear using a tool microscope. The experimental setup is shown in Figure 16.

**Figure 17:**
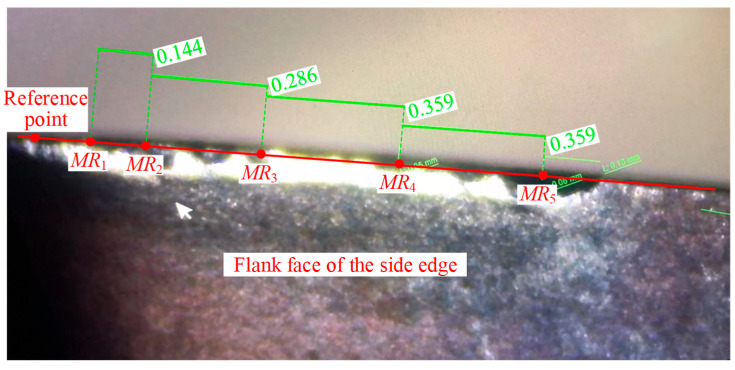
Measurement point positions for flank wear land width.

The experimental procedure was as follows:

1. **Inspecting the inserts:** A tool microscope was used to inspect the inserts, and only those without defects or damage were selected for use.
2. **Mounting and adjusting inserts:** Two inserts were installed onto the face milling tool. Each insert was marked with an oil-based marker as Insert 1 and Insert 2, respectively, for identification. The inserts were then manually adjusted to ensure that both axial and radial runout remained within 0.005 mm, and the actual tool radius was measured using an offline tool presetter.
3. **Workpiece setup:** The workpiece was clamped onto the machine tool table using a machine vise, and the workpiece coordinate system was aligned and set.
4. **Installing the tool and measuring tool length:** The tool was installed into the machine spindle. The tool length and radius were measured using a Renishaw non-contact laser tool setter. Specifically, five points on the tool bottom edge were selected for length measurement, labeled *ML*_1_ to *ML*_5_, with radial distances from the tool axis of 15.6, 15.4, 14.8, 14.4, and 14 mm, respectively.
5. **Measuring cutting edge radius:** For radius measurements, five height positions were defined: *MR*_1_ to *MR*_5_. Based on the maximum tool length, tool geometry, and cutting depth, the height levels were set at 0.1, 0.2, 0.4, 0.65, and 0.9 mm. Theoretically, *MR*_1_ and *MR*_2_ lie on the corner cutting edge, while *MR*_3_, *MR*_4_, and *MR*_5_ lie on the side cutting edge of the insert.
6. **Conducting the cutting experiment:** Face milling was performed in a reciprocating milling strategy to eliminate the effects of up and down milling. One complete back-and-forth pass was considered a single cutting segment, totaling a length of 220 mm. After completing one or more cutting segments as per the experimental design, cutting was paused. The tool length and radius were measured again using the same strategies in steps 4 and 5, and data were recorded.
7. **Tool wear image acquisition and flank wear measurement:** The tool was removed from the spindle, and wear images of the two inserts were captured using the tool microscope. Only the wear land on the flank face of the corner and side cutting edges was measured. The measurement heights corresponded to those in step 5. Since it was difficult to determine the tool end face and axis in the microscope, the side cutting edge was placed parallel to the microscope viewing plane, and the line of sight was aligned parallel to the rake face. Using the midpoint of the corner edge as a reference point, measurement positions along the side edge were offset accordingly. The calculated offset distances corresponding to the measurement heights were 0.144, 0.288, 0.574, 0.933, and 1.292 mm, with point intervals of 0.144, 0.286, 0.359, and 0.359 mm, respectively, as shown in Figure 17.
8. **Insert replacement decision:** Based on the tool wear condition, a decision was made whether to replace the insert. If chipping was observed, the insert was replaced, and the experiment resumed from step 2. If the insert remained valid, the experiment continued from step 4 until the tool was invalidated by chipping.

**Figure 18:**
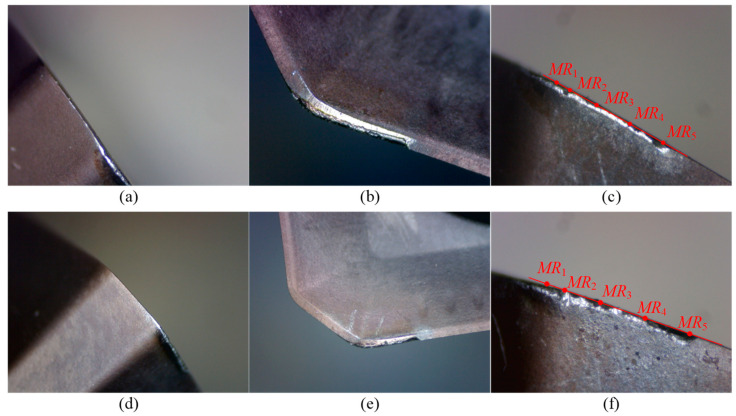
Wear images of Insert 1 of Tool 1. (**a**–**c**) show the wear conditions on the bottom flank, rake face, and side flank of the cutting edge, respectively, after two cutting segments; (**d**–**f**) show the corresponding wear conditions after four cutting segments.

**Figure 19:**
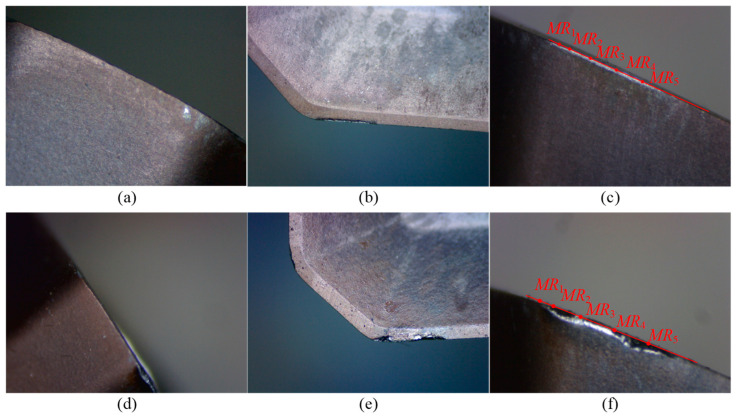
Wear images of Insert 2 of Tool 1. (**a**–**c**) show the wear conditions on the bottom flank, rake face, and side flank of the cutting edge, respectively, after two cutting segments; (**d**–**f**) show the corresponding wear conditions after four cutting segments.

**Figure 20:**
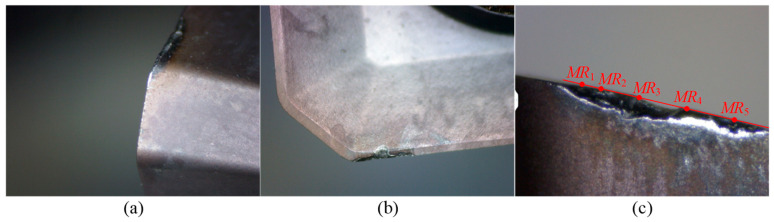
Wear images of Insert 1 of Tool 2. (**a**–**c**) show the wear conditions on the bottom flank, rake face, and side flank, respectively, after two cutting segments.

**Figure 21:**
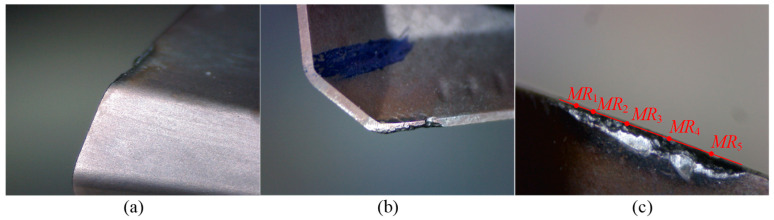
Wear images of Insert 2 of Tool 2. (**a**–**c**) show the wear conditions on the bottom flank, rake face, and side flank, respectively, after two cutting segments.

To determine the tool wear pattern and the height positions for radius measurement, cutting experiments are conducted following the steps described above. After completing two cutting segments, the tool is removed to capture flank wear images and measure flank wear land width. To enhance the stability of the experiment, two tools are used, denoted as Tool 1 and Tool 2. The experimental results are presented in Table 3 and Table 4, and Figure 18, Figure 19, Figure 20 and Figure 21.

For Tool 1, the tool radius is 16.054 mm. Table 3 presents the data of tool length, cutting edge radius, and flank wear land width for Tool 1. Figure 18 and Figure 19 show the wear images of Insert 1 and Insert 2 of Tool 1, including the wear conditions on the bottom flank, rake face, and side flank. From Table 3, with the increasing number of cutting segments, the variation in tool length remains within the range of 5 μm, indicating that wear on the bottom cutting edge is almost not worn. This is further supported by the images from the subgraphs (a) and (d) of Figure 18 and Figure 19, where the bottom edge wear appears very slight. In contrast, the variation in cutting edge radius reaches up to 70 μm, revealing significant wear on the corner and side cutting edges. Therefore, attention should be paid to flank wear on these edges. As shown in the subgraphs (c) and (f) of Figure 18 and Figure 19, flank wear on the corner and side cutting edges is more obvious. According to the flank wear data in Table 3, it can be seen that under the same cutting length, there are differences in wear amounts between the two inserts, as well as at different heights on the same insert. This indicates that during actual cutting, wear distribution is non-uniform both between inserts and along the height of a single insert. Hence, it is necessary to determine the location where wear failure is prone to occur through cutting experiments. Specifically, when the Insert 1 completes two and four cutting segments, the maximum wear occurs at point *MR*_5_, with wear amounts of 0.09 mm and 0.11 mm, respectively. The wear at point *MR*_3_ is the second highest. The point *MR*_1_ shows the smallest wear of 0.04 mm and 0.05 mm, respectively. In comparison, Insert 2 shows a more uniform wear distribution and the overall wear amount is relatively small, with a maximum wear of 0.10 mm, also occurring at point *MR*_5_.

Based on the wear conditions of the two inserts on Tool 1 (see Figure 18 and Figure 19), the primary wear pattern of the tool is flank wear on the corner and side cutting edges. Insert 1 shows more severe wear than Insert 2, possibly due to runout introduced during insert installation. Insert 1 has a larger radius, removes more material during cutting, and is subjected to greater cutting force, which leads to greater wear. In addition, after two cutting segments, both inserts remain in a normal wear state and can still continue cutting workpieces without any obvious clear characteristics of failure. However, after four cutting segments, chipping occurs on Insert 1 at point *MR*_5_, and chipping occurs on Insert 2 at point *MR*_5_ and a location somewhere between *MR*_2_ and *MR*_3_, indicating that the inserts are already in a failed state and can no longer continue effective cutting.

According to the analysis of Tool 1 above, it can be concluded that the two locations with the most severe wear and chipping are at points *MR*_3_ and *MR*_5_. Among them, point *MR*_5_ is located near the cutting depth, where a hardened layer may exist on the workpiece or its machined surface, resulting in increased stress and greater tool wear. Point *MR*_3_ is located on the side cutting edge near the corner cutting edge, which is the transition area between the two edges. Due to geometric discontinuity and stress concentration, this area is more prone to wear and chipping. Based on the above analysis, points *MR*_3_ and *MR*_5_ are identified as key positions for tool condition monitoring.

For Tool 2, its tool radius is 16.019 mm. Table 4 presents the data on tool length, cutting edge radius, and flank wear land width for Tool 2. Figure 20 and Figure 21 show the wear condition images of Insert 1 and Insert 2, respectively. Similarly to Tool 1, the variation in tool length for Tool 2 is also very small, indicating that there is almost no wear on the bottom cutting edge of the inserts. However, the change in cutting edge radius is relatively large, reaching 71 μm, which suggests that the corner cutting edge and side cutting edge experience significant wear. As shown in Table 4, it can be seen that Tool 2 has already experienced significant wear after only two cutting segments. Specifically, the maximum wear on Insert 1 occurs at point *MR*_5_, with a wear value of 0.11 mm. For Insert 2, the maximum wear appears at point *MR*_4_, also measuring 0.11 mm, while the wear amount at point *MR*_5_ is also relatively large, reaching 0.10 mm. In addition, from Figure 20 and Figure 21, obvious chipping is observed. Insert 1 shows chipping near point *MR*_3_, and Insert 2 exhibits chipping at point *MR*_5_, with the latter showing a larger chipped area.

Based on the previous experimental results, it has been identified the primary wear pattern of the tool is flank wear occurring on the corner cutting edge and side cutting edge. To further validate the accuracy of the proposed method for calculating the flank wear land width on these edges, and to determine the threshold value of the wear land width, two new inserts (referred to as Tool 3) were installed to conduct another cutting experiment following the same procedure as described above. In order to collect more experimental sample data before tool failure occurs, the tool is measured and wear images are carried out after each cutting segment, continuing until chipping failure of the inserts is observed. After completing the cutting experiment, the flank wear land widths are calculated using the proposed method in this study, based on the tool radius values measured by the laser tool setter at five different heights. The radius of Tool 3 is 16.004 mm, and the experimental results are presented in Table 5.

**Figure 22:**
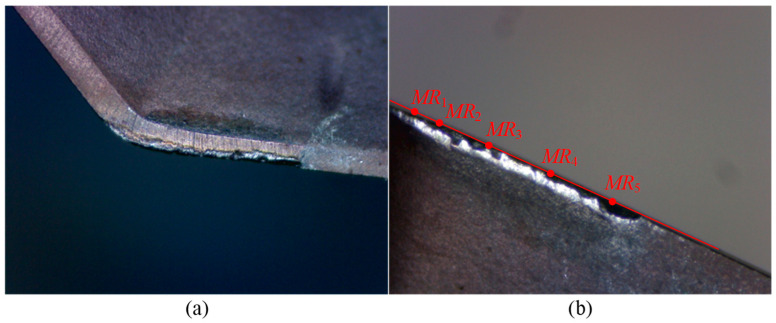
Wear images of Tool 3. (**a**,**b**) show the wear conditions on the rake face, and side flank, respectively, after four cutting segments.

Since the laser tool setter can only measure insert sizes with larger radii, the measured data recorded in Table 5 represent the wear values of inserts with greater wear. As shown in Table 5, Tool 3 experiences tool failure due to chipping after machining four cutting segments, with the chipping occurring at point *MR*_5_. The wear images of Tool 3 of the failed insert with chipping are shown in Figure 22. Furthermore, the calculated wear values exhibit good consistency with the measured wear values. After one cutting segment, the maximum error percentage is 14.00%, which occurs at measurement point *MR*_3_. After completing two, three, and four cutting segments, the maximum error percentages are 5.00%, 8.57%, and 3.33%, respectively, occurring at measurement points *MR*_1_, *MR*_2_, and *MR*_2_. These results indicate that the flank wear land width calculation method proposed in this study demonstrates high accuracy and provides reliable theoretical support for tool condition monitoring.

After completing three cutting segments, the maximum measured flank wear land width is 0.09 mm, and no chipping is observed on the tool. However, after four cutting segments, the tool fails due to chipping at point *MR*_5_. The measured wear values at the other four points are 0.07, 0.09, 0.09, and 0.09 mm, while the calculated wear values at the five measurement points are 0.070, 0.093, 0.092, 0.089, and 0.104 mm, respectively. Therefore, based on the above results, the threshold for flank wear land width is set to 0.10 mm to determine whether the tool is invalid.

In summary, through the cutting experiment of Experiment I, it was determined that the main wear pattern of the tool is flank wear on the corner and side cutting edges, and the failure form is mainly manifested as insert chipping. Based on wear characteristic analysis, the heights for measuring tool radius are set at the inflection point between the corner and side cutting edges, as well as near the cutting depth. In addition, the experimental data verified the effectiveness and high accuracy of the flank wear land width calculation method proposed in this study. At the same time, a critical wear threshold of 0.10 mm for flank wear land width is set to determine tool failure.

### 5.2. Experiment II

**Figure 23:**
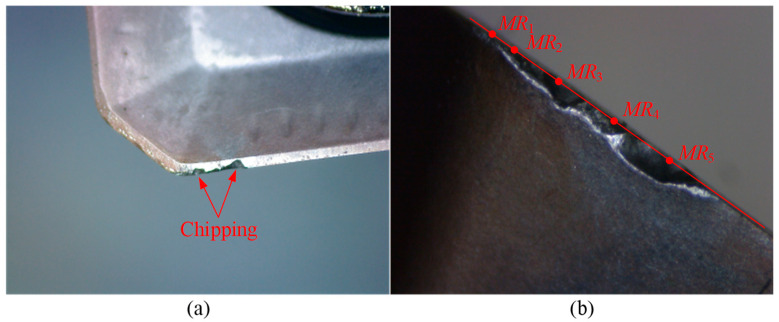
Wear images of Tool 4. (**a**,**b**) show the wear conditions on the rake face, and side flank, respectively, after four cutting segments.

Experiment II is conducted to verify the practicality of the tool condition monitoring method. A new tool, referred to as Tool 4, is prepared and the cutting process is performed following the same steps as in Experiment I. After completing two cutting segments, the laser tool setter is used to measure the tool radii at points *MR*_3_ and *MR*_5_. These radius values are substituted into the flank wear land width calculation method to obtain the calculated wear values. The calculated values are then compared with the predetermined critical wear threshold of 0.10 mm to determine whether the tool has failed. Additionally, the tool radius at point *MR*_5_ is updated in the tool list to compensate for the tool path. The radius of Tool 4 is 16.035 mm, and the experimental results are listed in Table 6, and the wear images are shown in Figure 23.

According to the experimental results in Table 6, after machining two cutting segments, the measured tool radii at points *MR*_3_ and *MR*_5_ are 16.355 mm and 16.830 mm, respectively. The corresponding calculated flank wear land widths are 0.069 mm and 0.076 mm, both of which are below the predetermined critical wear threshold of 0.10 mm. Therefore, the tool is considered valid and can continue machining. Before continuing with the machining, the tool radius is updated to 16.830 mm. After completing four cutting segments, the measured tool radii are 16.336 mm and 16.811 mm, and the calculated flank wear land widths are 0.102 mm and 0.110 mm, respectively. Both exceeded the predetermined critical wear threshold. As a result, the tool is determined to be in an invalid state. To confirm tool failure, the insert is placed under a tool microscope for wear image acquisition. As shown in Figure 23, severe chipping occurs at both points *MR*_3_ and *MR*_5_ on the larger worn insert. This result strongly demonstrates the practicality of the proposed tool condition monitoring method, which effectively predicts tool conditions and ensures machining quality.

The method proposed in this paper primarily involves a purely geometric calculation of the tool flank wear width based on the measured reduction in tool radius. The calculated wear value is then compared with a defined critical wear threshold. These calculations are minimal, requiring only a few milliseconds on a standard CNC controller or external PC. On our equipment (a Siemens 840D sl), the computation time for a single wear width calculation is approximately 20–30 milliseconds, negligible compared to the cycle time of a laser tool setter for tool dimension measurement (approximately 1–2 min). Therefore, this method can be applied in real time for on-machine tool condition monitoring without compromising machining efficiency. And, compared with the tool condition monitoring (TCM) method based on multi-sensor fusion and machine learning, the method proposed in this study has the following advantages. Specifically, (1) Unlike multi-sensor fusion systems (e.g., vibration, acoustic emission, and current), which require additional sensors hardware and complex signal processing, the proposed method leverages the existing on-machine laser tool setter, thus incurring no extra hardware cost. (2) The proposed method is deterministic and physically interpretable, eliminating the need for data-driven model training or large labeled datasets. (3) Integration into CNC systems is straightforward, as laser tool setters are already standard in many modern machine tools. Consequently, the proposed method does not rely on statistical learning, thereby avoiding issues such as overfitting or retraining when machining conditions change.

## 6. Conclusions

In the context of intelligent manufacturing, tool condition monitoring plays a critical role in achieving real-time monitoring of the machining process and improving machining accuracy and efficiency. The motivation of this study is to explore a method for calculating flank wear land width based on on-machine tool size measurement. Through theoretical analysis, geometric modeling, and cutting experiments, this research systematically investigates the formation mechanism of flank wear, a quantitative method for wear land width calculation, and the construction and validation of a tool condition monitoring approach. First, theoretical analysis reveals the mechanism of flank wear land formation. During the cutting process, the friction between the tool and the workpiece leads to progressive wear along the cutting edge, resulting in the development of a flank wear land, the width of which is closely related to the cutting edge radius of the tool. Then, a precise geometric model of the indexable face mill is established. Based on the geometric relationship between tool radius and flank wear land width, a calculation method for the flank wear land width that includes both the side and corner cutting edges is proposed, enabling quantitative calculation of the wear land width. Subsequently, a new tool wear measurement method is proposed based on the measurement capabilities of an on-machine laser tool setter. Finally, the accuracy and practicality of the proposed approach are verified by cutting experiments. Experimental results show that the primary wear pattern is flank wear on the corner and side cutting edges, and the dominant failure form is insert chipping. The optimized height of the tool radius measurement is set near the intersection point of the corner and side edges point *MR*_3_, as well as near the cutting depth point *MR*_5_. A wear land width threshold of 0.10 mm is established to determine tool failure. The proposed calculation method demonstrates high accuracy, with calculation errors controlled within 14.00%, satisfying the precision requirements of actual machining. Moreover, the proposed method for calculating the flank wear land width of indexable face mills can be extended to other types of cutting tools.

In future research, the calculated physical parameters such as flank wear width and tool radius variation could be integrated with machine learning algorithms to enable tool wear state classification, early failure prediction, and estimation of remaining useful life (RUL). By fusing physically interpretable measurements data with data-driven models, hybrid approaches could further enhance monitoring robustness and prediction accuracy of monitoring under different cutting conditions.

## Figures and Tables

**Table 1:** Geometric parameters of the indexable face milling tool and insert.

Number of Inserts, *N*_t_	Tool Radius, *R*_t_ (mm)		Axial Rake Angle, *θ*_a_ (°)	Radial Rake Angle, *θ*_r_ (°)
2	16		10	−15
Entering angle, *κ* (°)	Clearance angle, *α*_f_ (°)	Insert thickness, *TH*_in_ (mm)	Corner edge radius, *r*_c_ (mm)	Side edge length, *L*_se_ (mm)
45	20	4.76	1.5	9

**Table 2:** Cutting parameters in the experiment.

Spindle Speed, *n*_s_ (r/min)	Feed Rate, *f* (mm/min)	Axial Depth of Cut, *a*_p_ (mm)	Radial Width of Cut, *a*_e_ (mm)
1800	288	1	14

**Table 3:** Measurement results of Tool 1.

Insert Number	Number of Cutting Segments	Tool Length, *L*_t_ (mm)					Cutting Edge Radius, *R*_w_ (mm)					Flank Wear Land Width, *VB* (mm)				
		*ML*_1_	*ML*_2_	*ML*_3_	*ML*_4_	*ML*_5_	*MR*_1_	*MR*_2_	*MR*_3_	*MR*_4_	*MR*_5_	*MR*_1_	*MR*_2_	*MR*_3_	*MR*_4_	*MR*_5_
1	0	168.636	168.636	168.637	168.637	168.636	16.127	16.243	16.448	16.674	16.915	0	0	0	0	0
	2	168.634	168.633	168.635	168.634	168.634	16.096	16.198	16.406	16.635	16.865	0.04	0.07	0.08	0.07	0.09
	4	168.633	168.632	168.632	168.632	168.631	16.089	16.192	16.398	16.627	16.849	0.05	0.08	0.09	0.08	0.11
2	0	168.636	168.636	168.637	168.637	168.636	16.127	16.243	16.448	16.674	16.915	0	0	0	0	0
	2	168.632	168.633	168.635	168.634	168.634	16.096	16.198	16.406	16.635	16.865	0.01	0.01	0.03	0.03	0.03
	4	168.631	168.633	168.634	168.632	168.632	16.089	16.192	16.398	16.627	16.849	0.02	0.04	0.08	0.08	0.10

**Table 4:** Measurement results of Tool 2.

Insert Number	Number of Cutting Segments	Tool Length, *L*_t_ (mm)					Cutting Edge Radius, *R*_w_ (mm)					Flank Wear Land Width, *VB* (mm)				
		*ML*_1_	*ML*_2_	*ML*_3_	*ML*_4_	*ML*_5_	*MR*_1_	*MR*_2_	*MR*_3_	*MR*_4_	*MR*_5_	*MR*_1_	*MR*_2_	*MR*_3_	*MR*_4_	*MR*_5_
1	0	168.653	168.653	168.652	168.651	168.652	16.087	16.196	16.403	16.642	16.862	0	0	0	0	0
	2	168.652	168.651	168.651	168.650	168.650	16.052	16.154	16.340	16.598	16.791	0.05	0.07	0.11	0.06	0.07
2	0	168.653	168.653	168.652	168.651	168.652	16.087	16.196	16.403	16.642	16.862	0	0	0	0	0
	2	168.652	168.651	168.651	168.650	168.650	16.052	16.154	16.340	16.598	16.791	0.04	0.05	0.08	0.11	0.10

**Table 5:** Measurement results of Tool 3.

	Measurement Points	*MR*_1_	*MR*_2_	*MR*_3_	*MR*_4_	*MR*_5_
Number of Cutting Segments						
0	Initial Cutting Edge Radius, *R*_w_ (mm)	16.087	16.207	16.402	16.633	16.870
1	Cutting Edge Radius, *R*_w_ (mm)	16.067	16.183	16.377	16.618	16.850
	Calculated wear, *VB* (mm)	0.028	0.041	0.043	0.026	0.035
	Measured wear, *VB* (mm)	0.03	0.04	0.05	0.03	0.04
	Error percentage, (%)	−6.67	2.50	−14.00	−13.33	−12.50
2	Cutting Edge Radius, *R*_w_ (mm)	16.057	16.172	16.362	16.603	16.837
	Calculated wear, *VB* (mm)	0.042	0.059	0.069	0.052	0.058
	Measured wear, *VB* (mm)	0.04	0.06	0.07	0.05	0.06
	Error percentage, (%)	5.00	−1.67	−1.43	4.00	−3.33
3	Cutting Edge Radius, *R*_w_ (mm)	16.047	16.162	16.352	16.587	16.827
	Calculated wear, *VB* (mm)	0.056	0.076	0.087	0.080	0.076
	Measured wear, *VB* (mm)	0.06	0.07	0.09	0.08	0.08
	Error percentage, (%)	−6.67	8.57	−3.33	0.00	−5.00
4	Cutting Edge Radius, *R*_w_ (mm)	16.037	16.152	16.349	16.582	16.811
	Calculated wear, *VB* (mm)	0.070	0.093	0.092	0.089	0.104
	Measured wear, *VB* (mm)	0.07	0.09	0.09	0.09	Chipping
	Error percentage, (%)	0.00	3.33	2.22	−1.11	—

**Table 6:** Measurement results of Tool 4.

Number of Cutting Segments	0		2		4	
Measurement points	*MR*_3_	*MR*_5_	*MR*_3_	*MR*_5_	*MR*_3_	*MR*_5_
Cutting Edge Radius, *R*_w_ (mm)	16.410	16.881	16.355	16.830	16.336	16.811
Calculated wear, *VB* (mm)	0	0	0.069	0.076	0.102	0.110
Tool conditions	Valid	Valid	Valid	Valid	Invalid	Invalid

## Data Availability

The original contributions presented in this study are included in the article. Further inquiries can be directed to the corresponding authors.

## Nomenclature

**Symbol**	**Type**	**Description**
$X_{\mathrm{in}}Y_{\mathrm{in}}Z_{\mathrm{in}}O_{\mathrm{in}}$	Coordinate system	Insert coordinate system
$P_{\mathrm{se}}$	Scalar	Point on the side cutting edge of the insert
$P_{\mathrm{be}}$	Scalar	Point on the bottom cutting edge of the insert
$P_{\mathrm{ce}}$	Scalar	Point on the corner cutting edge of the insert
$O_{\mathrm{in}}$	Scalar	The intersection point of the side and bottom cutting edge
κ	Scalar	The entering angle of the insert (in radian)
$\alpha_{f}$	Scalar	The clearance angle of the insert (in radian)
$X_{t}Y_{t}Z_{t}O_{t}$	Coordinate system	Tool coordinate system
$\theta_{a}$	Scalar	The axial rake angle (in radian)
$\theta_{r}$	Scalar	The radial rake angle (in radian)
$R_{t}$	Scalar	The tool radius (in millimeters)
$L_{t}$	Scalar	The tool length (in millimeters)
$R_{w}$	Scalar	The cutting edge radius (in millimeters)
$M_{\mathrm{in}}^{t}$	Matrix	Homogeneous transformation matrix from $X_{\mathrm{in}}Y_{\mathrm{in}}Z_{\mathrm{in}}O_{\mathrm{in}}$ to $X_{t}Y_{t}Z_{t}O_{t}$
$N_{\mathrm{rf}}^{\mathrm{in}}$	Vector	Unit normal vector of the rake face in the insertion coordinate system
$N_{\mathrm{fse}}^{\mathrm{in}}$	Vector	Unit normal vector of the side edge flank face in the insertion coordinate system
$N_{\mathrm{fbe}}^{\mathrm{in}}$	Vector	Unit normal vector of the bottom edge flank face in the insertion coordinate system
$N_{\mathrm{rf}}^{t}$	Vector	Unit normal vector of the rake face in the tool coordinate system
$FS_{\mathrm{se}}^{\mathrm{in}}$	Function	The flank face of the side edge in the insert coordinate system
$FS_{\mathrm{se}}^{t}$	Function	The flank face of the side edge in the tool coordinate system
$h_{\mathrm{in}},d_{\mathrm{se}}$	Scalar	Height and width variable parameters (in millimeters)
$TH_{\mathrm{in}}$	Scalar	The thickness of the insert (in millimeters)
$r_{c}$	Scalar	The radius of the corner edge (in millimeters)
$L_{\mathrm{se}}$	Scalar	The length of the side edge (in millimeters)
$a_{p}$	Scalar	The axial depth of cut (in millimeters)
$a_{e}$	Scalar	The radial width of cut (in millimeters)
$n_{s}$	Scalar	The spindle speed (in r/min)
f	Scalar	The feed rate (in mm/min)
$PL_{\mathrm{se}}$	Scalar	The side edge flank wear plane
$P_{\mathrm{wse}}$	Scalar	The side edge wear point
$R_{\mathrm{wse}}$	Scalar	The radius of the cutting circle of the side edge (in millimeters)
$VB_{\mathrm{wse}}$	Scalar	The width of the wear land at side edge wear point $P_{\mathrm{wse}}$ (in millimeters)
$P_{\mathrm{sce}}$	Scalar	The tangent point of corner and side cutting edge
$P_{\mathrm{bce}}$	Scalar	The tangent point of corner and bottom cutting edge
$P_{\mathrm{ce}}^{\mathrm{in}}$	Function	The corner edge in the insert coordinate system
$\alpha_{\mathrm{ce}}$	Scalar	Angle variable parameter (in radian)
$l_{\mathrm{ce}}$	Scalar	Length variable parameter (in millimeters)
$V_{\mathrm{fce}}$	Vector	The direction of the intersection line between the flank face of the side and the bottom edge
$FS_{\mathrm{ce}}^{\mathrm{in}}$	Function	The flank face of the corner edge in the insert coordinate system
$FS_{\mathrm{ce}}^{t}$	Function	The flank face of the corner edge in the tool coordinate system
$P_{\mathrm{wce}}$	Scalar	The corner edge wear point
$R_{\mathrm{wce}}$	Scalar	The radius of the cutting circle of the corner edge (in millimeters)
$VB_{\mathrm{wce}}$	Scalar	The width of the wear land at corner edge wear point $P_{\mathrm{wce}}$ (in millimeters)
$N_{t}$	Scalar	Number of inserts
$ML_{i}$	Scalar	The *i*-th length measurement point
$MR_{i}$	Scalar	The *i*-th radius measurement point
